# Recent developments in the use of aza-Heck cyclizations for the synthesis of chiral N-heterocycles

**DOI:** 10.1039/c7sc01480e

**Published:** 2017-06-20

**Authors:** Nicholas J. Race, Ian R. Hazelden, Adele Faulkner, John F. Bower

**Affiliations:** a School of Chemistry , University of Bristol , Bristol , BS8 1TS , UK . Email: john.bower@bris.ac.uk

## Abstract

Aza-Heck cyclizations are an emerging method for the construction of chiral N-heterocyclic systems. In these processes, an activated N–O bond replaces the C–X bond (X = halide, OTf) used in conventional Heck reactions, with the associated aza-Pd(ii)-intermediate engaging pendant alkenes in a Heck-like manner. This perspective article commences with an historical overview of the area, which stems from Narasaka's seminal studies using oxime esters as the initiating motif. The scope and mechanism of associated chiral N-heterocyclic methodologies are then outlined, including cascade processes that enable diverse alkene 1,2-carboaminations. The recent emergence of new N–O donors and the realization of highly enantioselective aza-Heck cyclizations are then discussed. Collectively, these studies suggest that the aza-Heck approach can underpin a broad family of redox-neutral and enantioselective C–N bond forming processes.

## Abstract 2

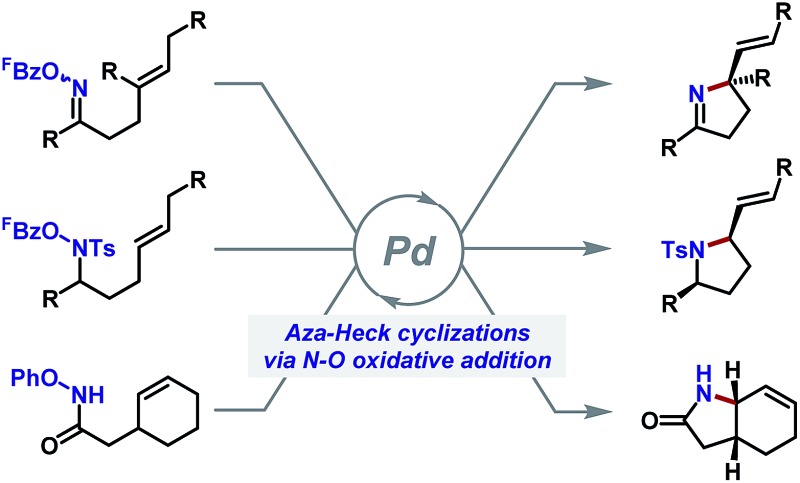
The scope and mechanism of aza-Heck methodologies that provide chiral heterocyclic systems are outlined.

## Introduction

Nitrogen-containing molecules hold a privileged position in drug discovery. In 2013, eighty percent of the top twenty best-selling brand name drugs incorporated nitrogen, with two thirds of these compounds containing an N-heterocycle.^1^ In particular, chiral pyrrolidine and piperidine derivatives are common in pharmaceuticals and alkaloid natural products.^2^ With these considerations in mind, the development of efficient and versatile routes to complex molecular structures, particularly those containing C(sp^3^)–N bonds, is of paramount importance.^3^

**Scheme 1:**
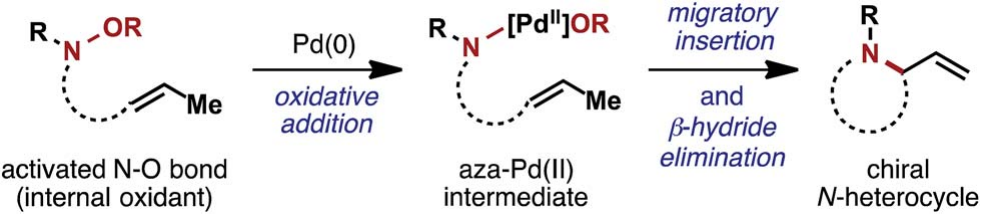
Synthesis of chiral N-heterocycles by aza-Heck cyclization.

In this perspective, we highlight the emerging area of aza-Heck reactions for the synthesis of chiral nitrogen heterocycles. Here, an activated N–O bond replaces the C–X bond (X = halide, OTf) used in conventional Heck reactions, with the resulting aza-Pd(ii) intermediates mimicking the reactivity of their aryl-Pd(ii) counterparts (Scheme 1). In general terms, aza-Heck reactions can be considered complementary to more well-established aza-Wacker processes that require an external oxidant.^4^ However, as will be seen, the use of an N–O bond as the internal oxidant can confer several synthetic and mechanistic advantages. The following discussion highlights key historical developments in aza-Heck chemistry before focussing on the scope and mechanism of methodologies that provide chiral heterocyclic systems.

## The Narasaka–Heck reaction and early heteroaromatic methodologies

**Scheme 2:**
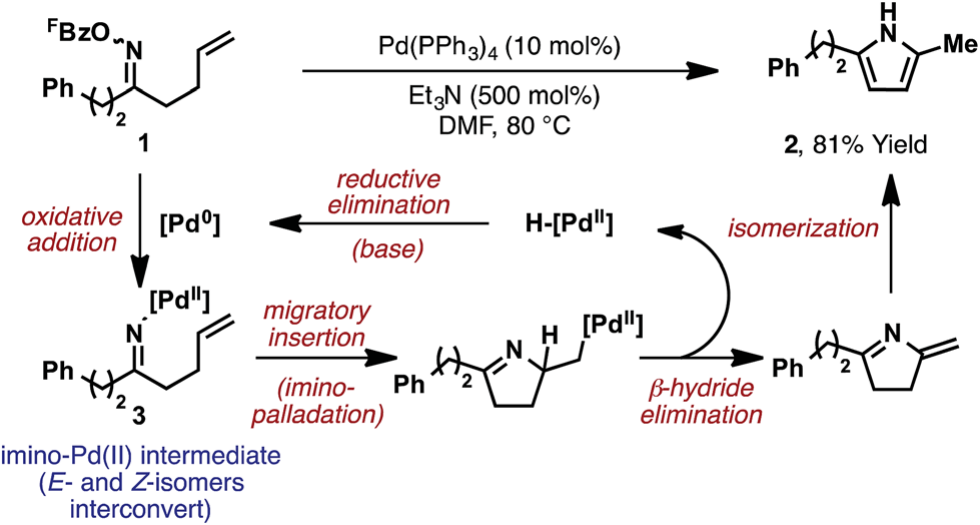
The Narasaka–Heck reaction.

The prototypical aza-Heck process was reported in 1999 by Narasaka and co-workers. In this seminal work, exposure of γ,δ-unsaturated pentafluorobenzoyl oxime ester **1** (^F^Bz = pentafluorobenzoyl) to 10 mol% Pd(PPh_3_)_4_ effected cyclization to pyrrole **2** in 81% yield (Scheme 2).^5^ A variety of other systems were shown to cyclize efficiently, providing a direct and flexible entry to differentially substituted pyrroles. The proposed mechanism involves elementary steps that are analogous to the conventional Heck reaction,^6^ with the process later becoming known as the ‘Narasaka–Heck reaction’.^7^ Specifically, oxidative addition of Pd(0) into the N–O bond of **1** generates key imino-Pd(ii) intermediate **3**; this initiation step replaces the C–X oxidative addition step of the conventional Heck reaction. From **3**, migratory insertion of the alkene into the Pd–N bond (5-*exo* imino-palladation)^8^ is followed by β-hydride elimination and alkene isomerization to deliver the pyrrole target. Importantly, the geometry of the oxime ester moiety of **1** is inconsequential, such that both isomers cyclize with comparable efficiencies. Presumably, *E*/*Z* isomerization occurs at the stage of imino-Pd(ii) intermediate **3**, although the mechanism of this process is not well understood. Imino-Pd(ii) intermediates related to **3** have been characterized crystallographically by the groups of Hartwig^9^ and Stahl,^10^ providing unambiguous confirmation of the N–O oxidative addition event.

**Scheme 3:**
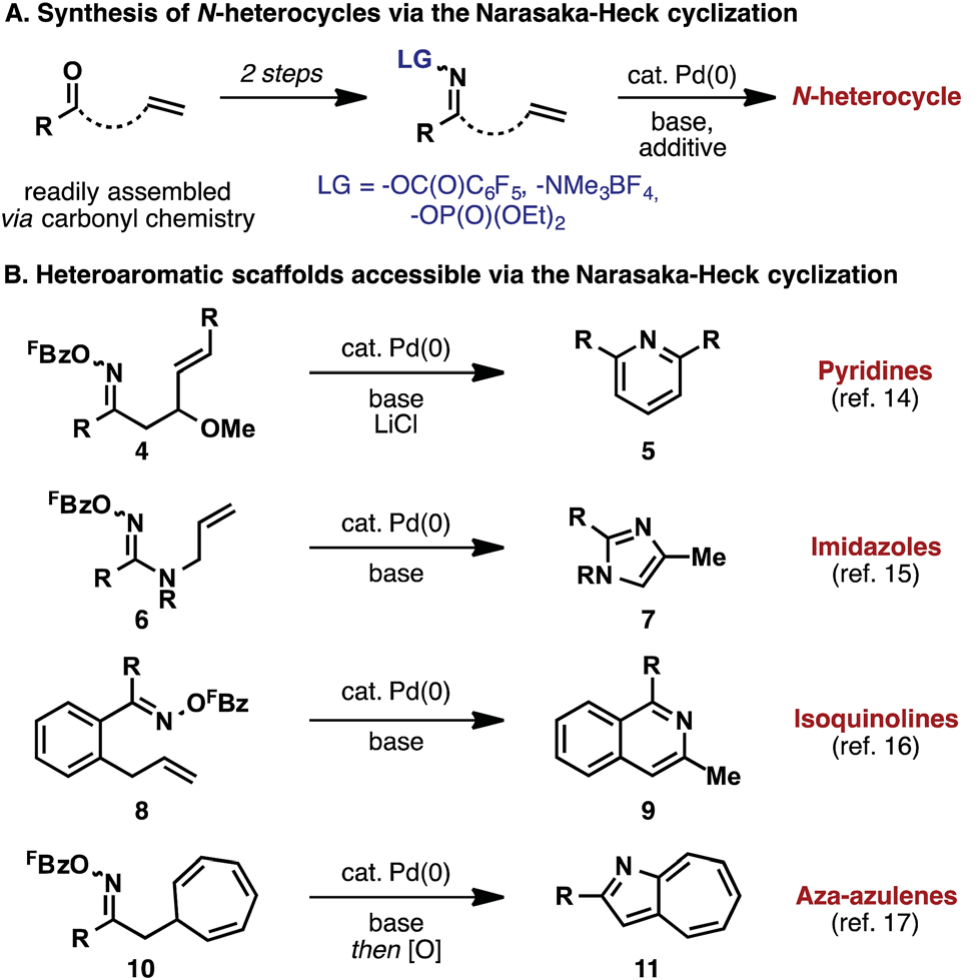
Heterocycle synthesis *via* the Narasaka–Heck reaction.

Following Narasaka's initial report, a variety of related heteroaromatic methodologies emerged (Scheme 3). A key feature of all of these approaches is that the oxime ester substrates are readily available from the corresponding ketones, which, in turn, can be accessed using well-established carbonyl chemistry (Scheme 3A). In most cases, *O*-pentafluorobenzoyl oxime esters are required, although processes that exploit oxidative addition into the N–O bond of *O*-phosphinyl oxime esters^11^ or the N–N bond of *N*,*N*,*N*-trimethylhydrazonium salts have also been developed.^12^ *N*-Chloroamines have also been suggested as suitable initiating motifs, although subsequent studies suggest that cyclization of such systems proceeds *via* a radical-based pathway.^13^

A representative outline of heteroaromatic compounds accessible *via* the Narasaka–Heck reaction is presented in Scheme 3B. Pyridines **5** can be accessed by 6-*endo* cyclization of oxime esters **4**; here, chloride additives such as LiCl and *n*-Bu_4_NCl were found to play a crucial role.^14^ Abell and co-workers showed that amidoxime esters **6** cyclize to afford imidazoles **7**, thereby demonstrating the viability of N–O oxidative addition beyond oxime ester substrates.^15^ In conformationally constrained cases, 6-*exo* cyclizations are feasible; for example, isoquinolines **9** can be accessed by aza-Heck cyclization of precursors **8**.^16^ Other classes of acceptor can also be exploited. For example, cyclization onto pendant cycloheptatrienes generates aza-azulenes after *in situ* oxidation of the initial aza-Heck adduct (**10** to **11**).^17^

**Scheme 4:**
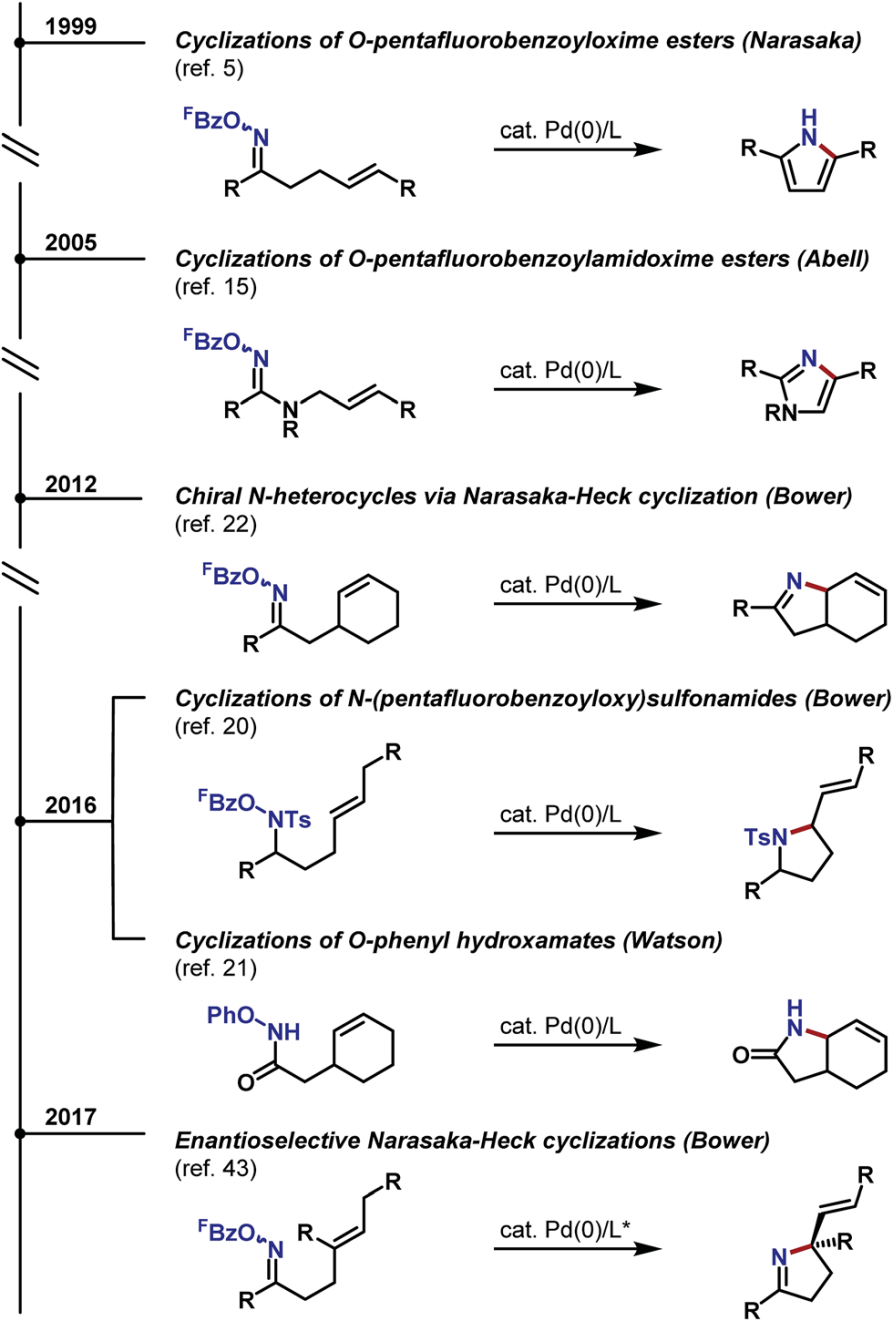
Historical overview of key developments in aza-Heck reactions.

The processes discussed so far use the Narasaka–Heck reaction to generate new C(sp^2^)–N bonds,^18^ rendering these approaches ideally suited to the synthesis of N-heteroaromatic compounds. Given the increasing importance of chiral N-heterocycles in the pharmaceutical industry,^2^ generalization of aza-Heck manifolds to the formation of (stereodefined) C(sp^3^)–N bonds is particularly appealing. The remainder of this Perspective highlights recent aza-Heck methodologies that use N–O bonds to generate chiral nitrogen heterocycles.^19^ As will be seen, processes that initiate at the N–O bond of oxime esters, as used in the original Narasaka process, have received the most attention. However, recent reports have exploited other redox active N–O donors, such that *N*-(pentafluorobenzoyloxy)sulfonamide^20^ and *O*-phenyl hydroxamates^21^ are now viable initiating motifs for aza-Heck cyclizations (Scheme 4).

## Chiral N-heterocycles *via* Narasaka–Heck cyclizations: mechanism and reaction scope

**Scheme 5:**
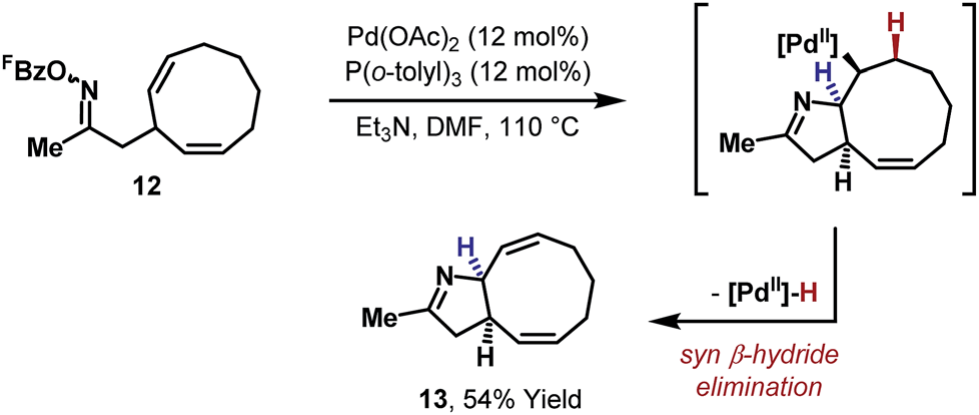
Fürstner's use of an aza-Heck cyclization onto a cyclic alkene.

In 2012, the first systematic efforts to exploit aza-Heck cyclizations of oxime esters for the synthesis of C(sp^3^)–N bonds, and therefore chiral N-heterocycles, were reported by our group. Initial studies explored cyclizations onto cyclic alkenes because the stereoelectronic requirements of *syn*-iminopalladation and *syn*-β-hydride elimination were expected to result in the formation of C(sp^3^)–N bonds.^22^ Indeed, an example of this type of cyclization had been reported in 2005 by Fürstner and co-workers in the context of a natural product synthesis (Scheme 5).^23^ In this process, a symmetrical bis-olefinic acceptor unit was specifically incorporated into **12** to offset inherent inefficiencies associated with imino-palladation. Even using this biasing factor, high loadings of Pd catalyst were required to achieve a relatively modest yield of target **13**. At the time, these issues were reflective of wider difficulties in promoting efficient aza-Heck cyclizations over diverse substrate classes.

**Scheme 6:**
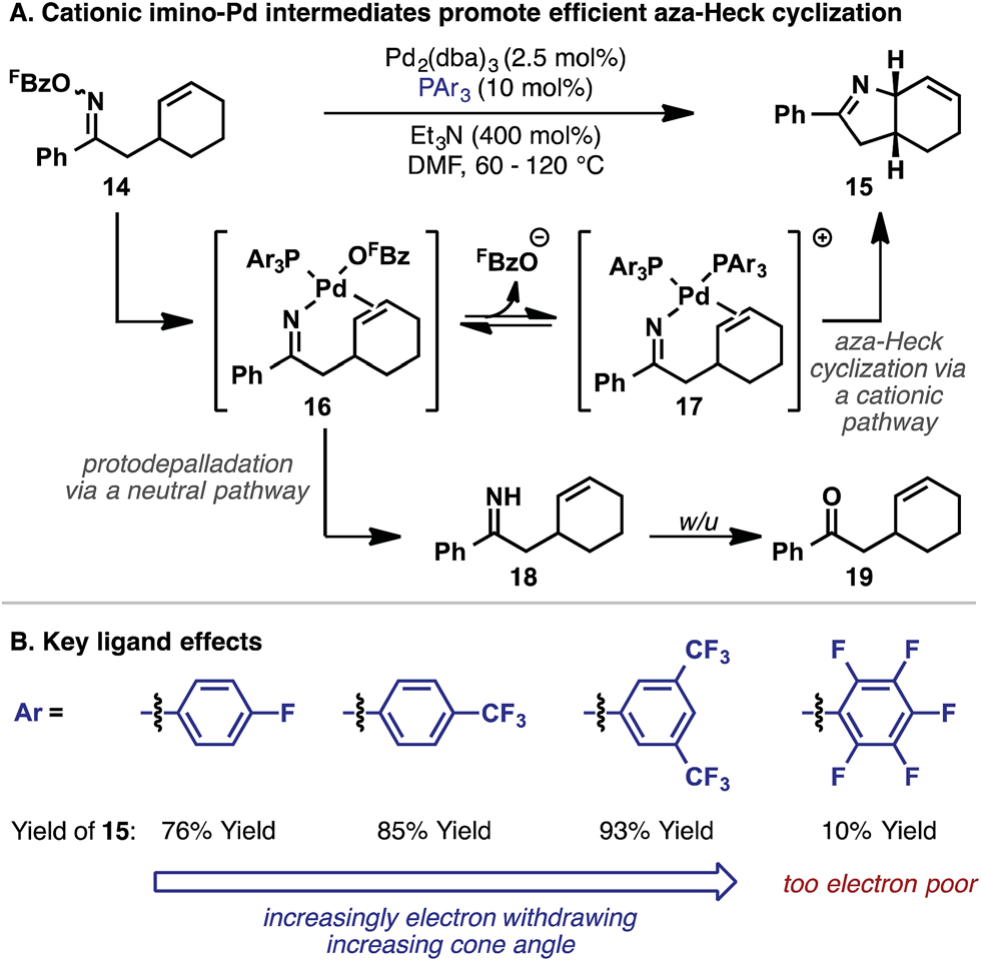
Key features of the aza-Heck cyclization of *O*-pentafluorobenzoyl oxime esters.

With the aim of developing a general aza-Heck methodology involving cyclic alkenes, oxime ester **14** was used as a model substrate (Scheme 6A).^22^ Extensive investigations revealed that efficient cyclization to **15** was possible, but only when electron-deficient phosphine ligands were used. By far the most effective subset of ligands were fluorinated triarylphosphines, with P(3,5-(CF_3_)_2_C_6_H_3_)_3_ emerging as the optimal system (Scheme 6B). This ligand allowed cyclization of **14** to proceed in 93% yield at 60 °C with 5 mol% Pd loading. Highly electron-deficient phosphine ligands, such as P(C_6_F_5_)_3_ were not effective and low conversions were observed, likely due to slow N–O oxidative addition. However, within the effective ligand range outlined in Scheme 6B, oxidative addition does not appear to be rate limiting such that the choice of ligand is dictated by the efficiency of steps later in the cycle. Interestingly, bulky electron-rich phosphine ligands, such as those typically employed in conventional Heck reactions (where oxidative addition is often rate limiting), are not effective and lead to a different mechanistic pathway (*vide infra*).

The beneficial effects of P(3,5-(CF_3_)_2_C_6_H_3_)_3_ are consistent with stoichiometric studies by Hartwig and co-workers, who demonstrated that migratory insertion of alkenes into Pd-NPh_2_ bonds is facilitated by bulky and/or electron-deficient ligands.^24^ An additional key factor for the conversion **14** to **15** resides in suppression of protodepalladation of imino-Pd intermediate **16**. Electron-deficient phosphine ligands are expected to increase σ-donation from the imino moiety, which, in turn, should suppress competing protonation of this unit. Indeed, under unoptimized conditions, protodepalladation dominates, leading to ketone by-product **19**, likely *via* the intermediacy of hydrolytically sensitive NH imine **18**.

Also of considerable importance for the cyclization of **14** to **15** is the pentafluorobenzoate leaving group. This activating group was introduced in Narasaka's seminal publication as a result of empirically driven studies;^5^ substrates of this type were found to offer the best balance of starting material stability and catalytic activity. With respect to the former aspect, the key issue is suppression of competing Beckmann rearrangement of the starting material, which becomes problematic when more polarizing activating groups (*e.g. O*-sulfonyl groups) are used. Nevertheless, the requirement of a pentafluorobenzoate leaving group for the cyclization of **14** was striking given that Uemura and co-workers have shown that facile oxidative addition of Pd(0) into the N–O bond of less polarized *O*-benzoyl oximes can be used to trigger β-carbon eliminations.^25^ Note that the Uemura process does not involve alkene imino-palladation, suggesting a critical role for the leaving group on the efficiency of this step in aza-Heck reactions.

**Scheme 7:**
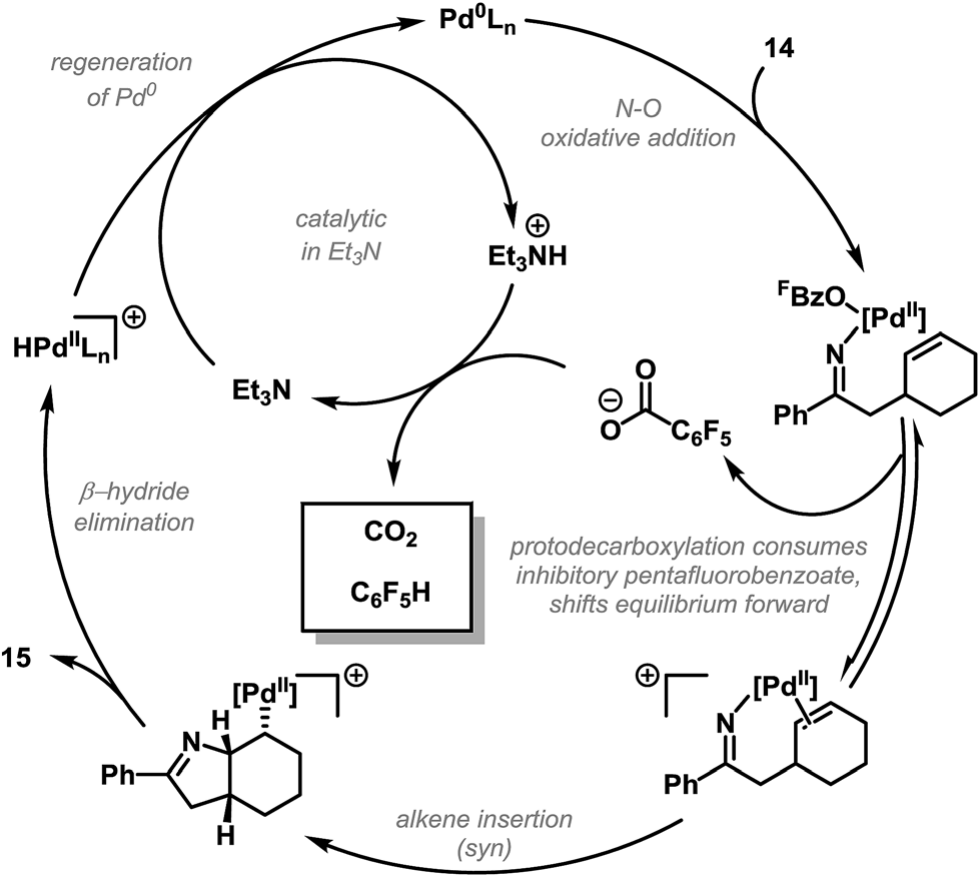
Detailed catalytic cycle of the aza-Heck cyclization of oxime ester **14**.

Investigations aimed at understanding the efficiencies of different leaving groups (in particular pentafluorobenzoate *vs.* benzoate) revealed that, for the cyclization of **14** to **15**, addition of exogenous C_6_F_5_CO_2_H or C_6_H_5_CO_2_H decreases both the reaction rate and yield.^26^ Furthermore, halide additives, such as *n*-Bu_4_NCl, completely suppress aza-Heck cyclization. These studies suggest that, for efficient cyclization, the leaving group must dissociate after oxidative addition to provide cationic imino-Pd(ii) intermediate **17** (Scheme 6A). Relatively electron-rich carboxylates, such as benzoate, do not dissociate readily, resulting in less efficient cyclization of **14** and increased levels of protodepalladation at the stage of imino-Pd(ii) intermediate **16**. ^19^F NMR studies revealed a surprising additional benefit of the pentafluorobenzoate leaving group. It was found the interaction of this by-product with protonated triethylamine, which is generated by deprotonation of the Pd-hydride species released upon β-hydride elimination, results in facile protodecarboxylation to C_6_F_5_H. This process removes otherwise inhibitory pentafluorobenzoate from the reaction system, such that equilibrium access to cationic imino-Pd(ii) intermediate **17** is maintained in subsequent cycles. In favorable cases, the protodecarboxylation process allows substoichiometric (catalytic) quantities of Et_3_N to be used. Thus, efficient aza-Heck cyclization is dependent on both a pentafluorobenzoate leaving group and an electron-deficient phosphine. A complete mechanistic picture of the aza-Heck cyclization of **14** to **15**, as discussed above, is outlined in Scheme 7.

**Scheme 8:**
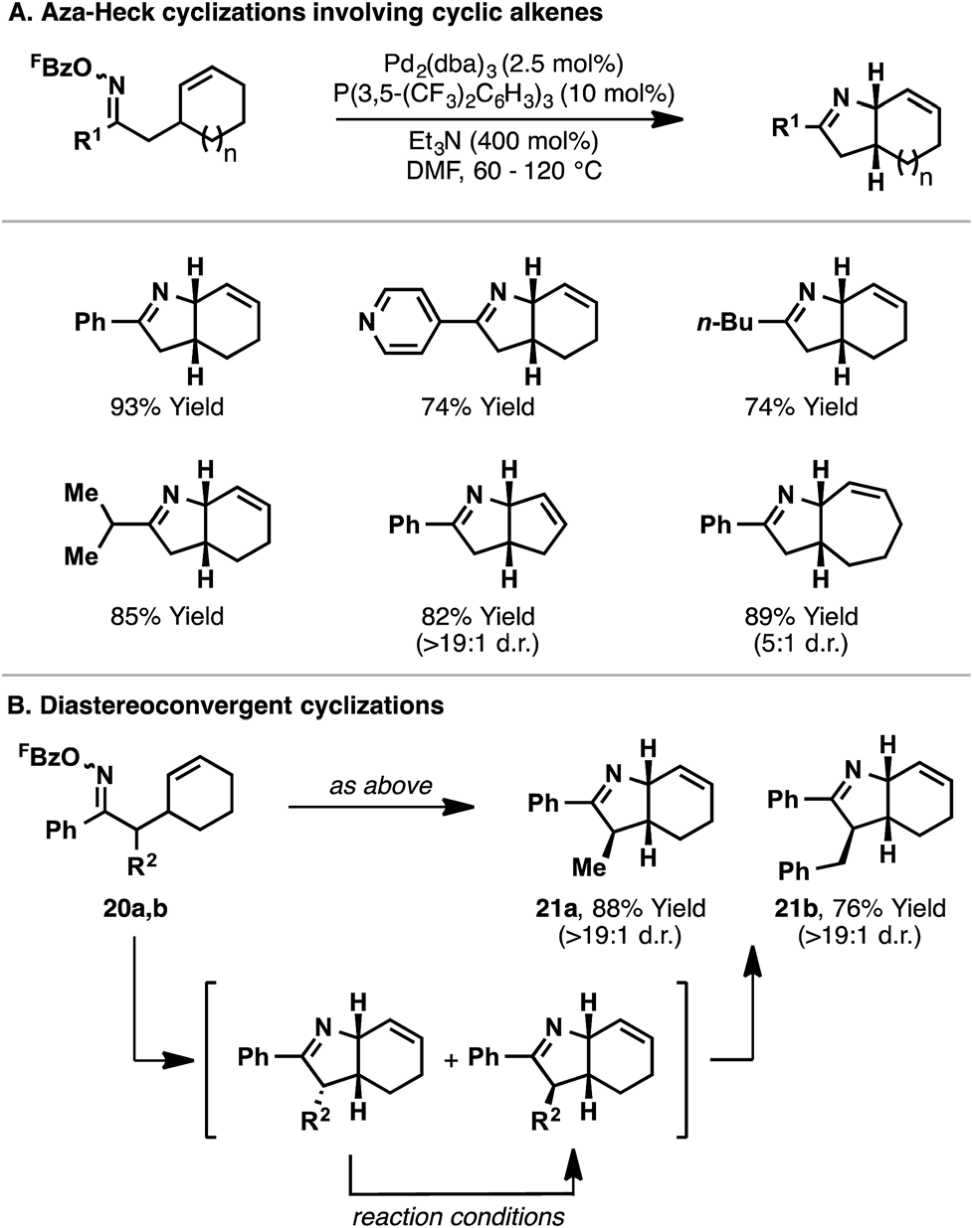
Aza-Heck cyclizations of oxime esters with cyclic alkenes.

The conditions optimized for **14** proved to be generally applicable for 5-*exo* aza-Heck cyclizations onto cyclic alkenes (Scheme 8).^22^ Indeed, a variety of aryl-, alkyl-, and heteroaryl-substituted oxime esters underwent efficient aza-Heck reaction to give the corresponding *cis*-fused bicyclic N-heterocycles in high yield (Scheme 8A). Cyclic alkenes of different ring sizes were also tolerated, as well as systems with substitution at C2 of the oxime ester. Interestingly, oxime ester **20a**, which possesses a non-defined α-stereocenter, underwent stereoconvergent cyclization to generate bicyclic system **21a** in high diastereopurity; a similar result was observed for **21b** (Scheme 8B). Presumably, initial cyclization of both diastereomers is followed by equilibration (*via* the enamine) to the thermodynamically favored product, wherein the R^2^-substituent resides on the less hindered face of the dihydropyrrole ring.

**Scheme 9:**
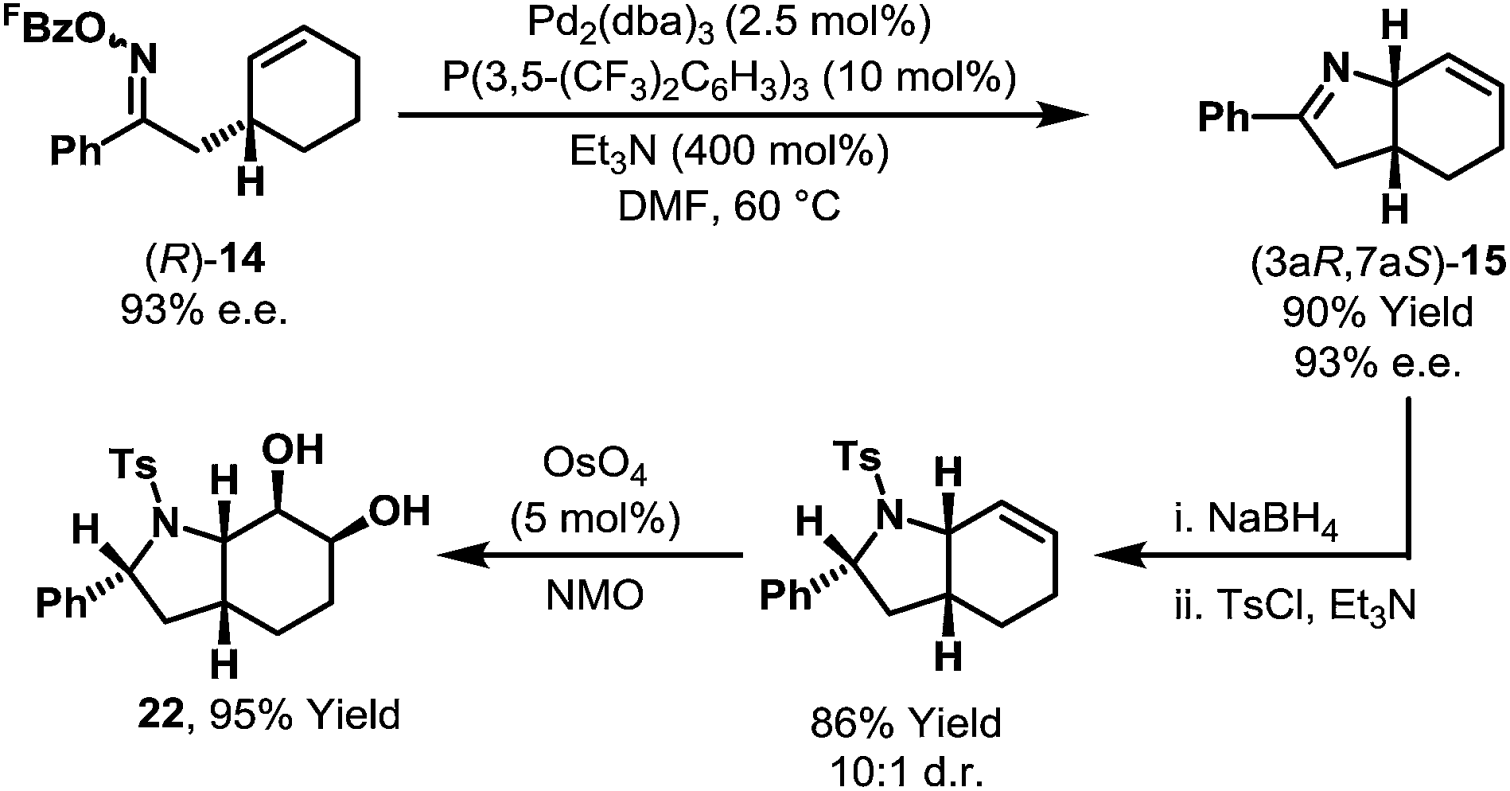
Synthesis of a complex, chiral N-heterocycle using aza-Heck methodology.

An inherent consequence of using the Narasaka–Heck cyclization to generate C(sp^3^)–N bonds is that the product contains imine and alkene units for further derivatization. Oxime ester (*R*)-**14** (93% e.e.) underwent diastereoselective aza-Heck cyclization to afford N-heterocycle (3a*R*,7a*S*)-**15** without loss of enantiomeric purity (Scheme 9). Further manipulation of this product *via* the imine and alkene afforded complex bicyclic system **22** with high levels of control for all five stereocenters. The sequence in Scheme 9 demonstrates the power of aza-Heck methodology for the formation of complex, chiral N-heterocycles.

**Scheme 10:**
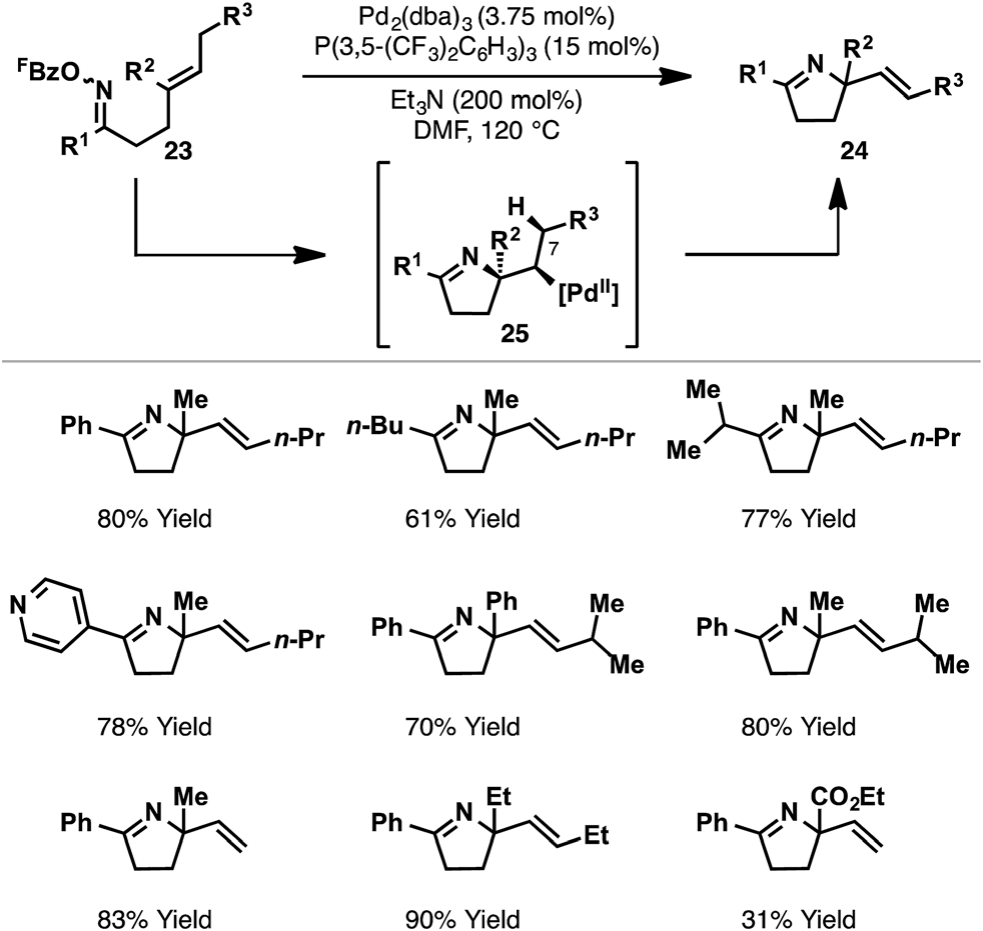
Aza-Heck cyclization of oxime esters with 1,1-disubstituted alkenes.

Further studies sought to investigate the scope of the Pd_2_(dba)_3_/P(3,5-(CF_3_)_2_C_6_H_3_)_3_ catalyst system with respect to other classes of alkene acceptor. It was found that cyclizations of oxime esters with 1,1-disubstituted alkenes (Scheme 10) provides a general entry to α,α-disubstituted systems **24**.^27^ Here, 5-*exo* cyclization of **23** generates alkyl-Pd(ii) intermediate **25**, which can only undergo β-hydride elimination *via* C7–H to deliver chiral product **24**. The process tolerates sterically and electronically diverse substituents at R^1^ and R^2^, providing an efficient approach to challenging tetra-substituted nitrogen-bearing stereocentres. Ultimately, this study served as the basis for the development of enantioselective variants, as described later.

**Scheme 11:**
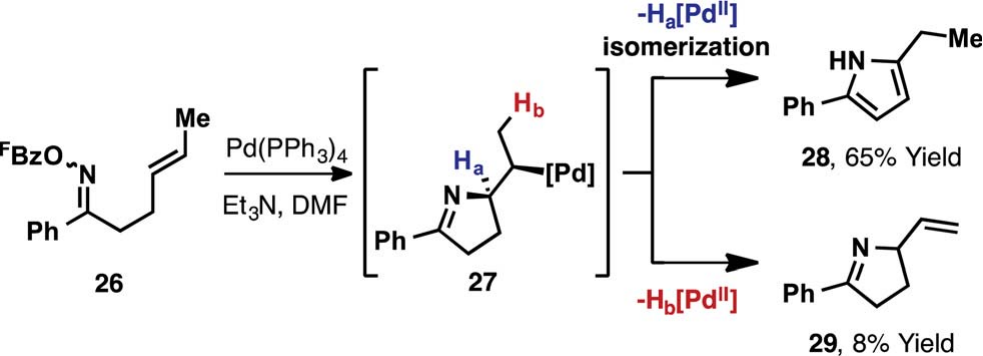
β-Hydride elimination pathways in aza-Heck cyclizations with 1,2-disubstituted alkenes.

In the studies outlined above, which involve cyclic or 1,1-disubstituted alkenes, the nature of the alkene acceptor enforces the formation of chiral products (new C(sp^3^)–N bond). For processes involving 1,2-disubstituted alkenes, 5-*exo* cyclization generates alkyl-Pd(ii) intermediate **27**, from which β-hydride elimination can occur *via* either H_a_ or H_b_ (Scheme 11). The former pathway leads to pyrrole products; Narasaka has shown that cyclisation of **26** using Pd(PPh_3_)_4_ affords pyrrole **28** with 8 : 1 selectivity over dihydropyrrole **29** (73% combined yield).^5^

**Scheme 12:**
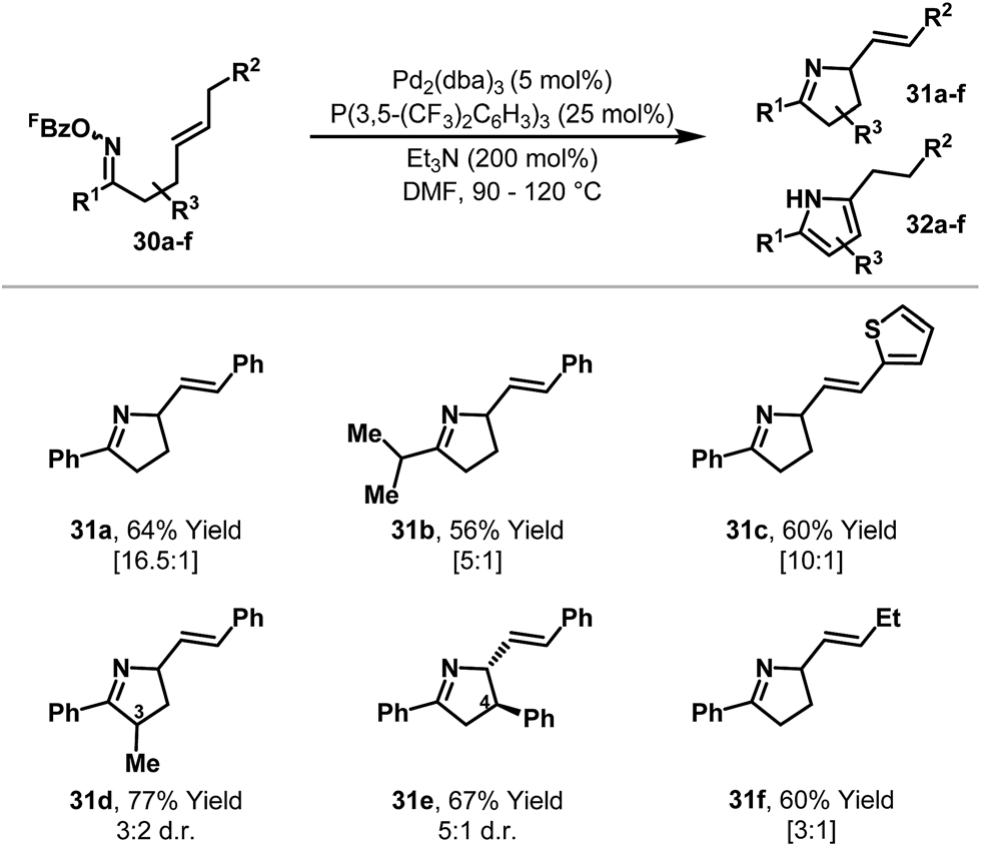
Aza-Heck cyclizations of oxime esters with 1,2-disubstituted alkenes; the ratio of dihydropyrrole **31** : pyrrole **32** is shown in brackets below the isolated yield of pure **31**.

To provide selectivity for chiral products in processes involving 1,2-disubstituted alkenes, strategic installation of an aryl ring at R^2^ was explored (Scheme 12).^28,29^ This approach proved effective, such that a variety of dihydropyrroles **31a–e** were generated from oxime esters **30a–e** with high selectivity over the corresponding pyrroles **32a–e**. For systems with substitution at C4 (*e.g.* **31e**) good levels of 1,2-diastereocontrol were observed, whereas C3-methyl substituted product **31d** was formed in low diastereoselectivity. Gratifyingly, extension of the method to systems where R^2^ = alkyl also resulted in selective formation of chiral products (*e.g.* **30f** to **31f**). Here, studies on the effects of different ligands revealed that the relatively wide cone angle of P(3,5-(CF_3_)_2_C_6_H_3_)_3_ is responsible for preferential β-hydride elimination *via* H_b_.

**Scheme 13:**
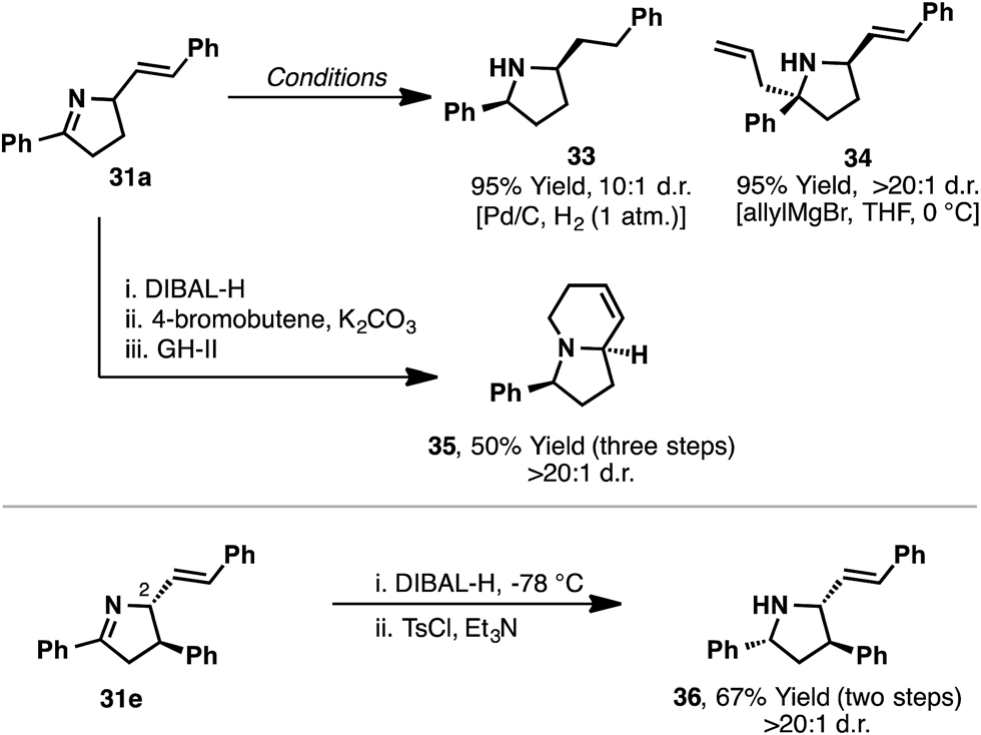
Chiral N-heterocycles accessible from dihydropyrrole product.

The dihydropyrrole products accessed in Scheme 12 are ideally suited to diastereoselective manipulations (Scheme 13). Indeed, hydrogenation of the imine of **31a** afforded pyrrolidine **33** in 95% yield and 10 : 1 d.r. Similarly, allyl addition (allylMgCl) to **31a** occurred from the less hindered face of the imine to provide **34** in high diastereoselectivity (>20 : 1 d.r.). Bicyclic systems common to many indolizidine alkaloids can also be constructed. For example, DIBAL-H reduction of the imine of **31a** was followed by *N*-butenylation and ring-closing metathesis to afford bicyclic system **35** in high diastereopurity. More stereochemically complex aza-Heck products can also be manipulated, with DIBAL-H reduction of **31e** occurring from the face opposite the C2-substituent to provide complex pyrrolidine **36** in >20 : 1 d.r.

## Imino-palladation *versus* iminyl radical cyclization in Narasaka–Heck reactions and a copper-catalyzed protocol

**Scheme 14:**
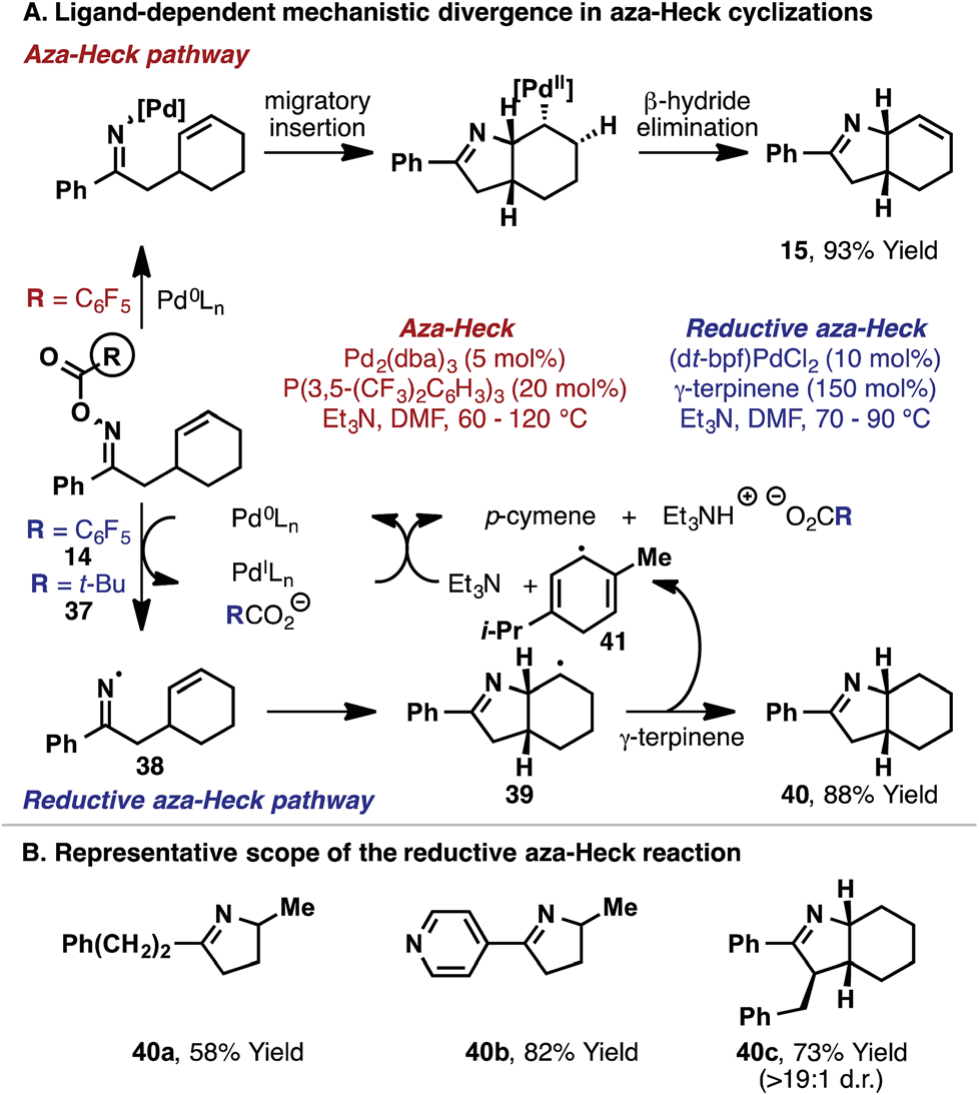
Reductive aza-Heck reaction *via* iminyl radical cyclization.

The studies described so far highlight the importance of electron-deficient ligands for efficient aza-Heck cyclization. In part, this may be due to an enhancement of alkene migratory insertion at the stage of the imino-Pd(ii) intermediate. However, another key benefit resides in the mechanistic control that such ligands offer for the N–O oxidative addition step. In early studies, we found that when common electron-rich ligand systems (*e.g.* 1,1′-bis(di-*tert*-butylphosphino)ferrocene (d*t*-bpf), P(*t*-Bu)_3_, PCy_3_ *etc.*) were used for cyclization of oxime ester **14** or pivaloyl variant **37**, aza-Heck product **15** was not generated and instead saturated system **40** formed (Scheme 14).^30^ Although **40** is formally a reductive aza-Heck product, a series of mechanistic experiments support an alternate radical-based reaction pathway. Indeed, the yield of **40** is enhanced by the inclusion of common hydrogen atom donors, such as γ-terpinene, whereas hydride donors offer no appreciable benefit. Our collective studies suggest that single-electron transfer from the electron-rich Pd(0)-catalyst to **14** or **37** forms iminyl radical **38**, with concomitant generation of Pd(i) and carboxylate anion. Fast 5-*exo* cyclization then affords alkyl radical **39**, which can abstract a hydrogen atom from γ-terpinene to generate ‘reductive’ aza-Heck product **40** and bis-allylic radical **41**. Hydrogen-atom transfer from **41** to Pd(i) affords *p*-cymene and a Pd(ii)-hydride, which undergoes base-promoted reductive elimination to regenerate the Pd(0)-catalyst. The process outlined in Scheme 14A is generally applicable, with the unusual hybrid organometallic-radical protocol^31^ providing an appealing entry to iminyl radical chemistry.^32^ Indeed, products **40a–c** were all accessed efficiently using this approach (Scheme 14B).

Overall, the ligand effects revealed during our studies highlight important factors that may impact other processes involving oxidative addition of Pd(0) into the N–O bond of oxime esters. Specifically, it appears that electron-neutral or electron-poor Pd(0)-systems undergo N–O oxidative addition *via* a concerted two-electron redox process, whereas electron-rich Pd-systems can deviate into a SET pathway. For the process in Scheme 14, this pathway is interrupted by fast cyclization of the iminyl radical onto the pendant alkene. In systems without an alkene acceptor, stoichiometric studies from the groups of Hartwig^9^ and Stahl^10^ have shown that electron-rich Pd-systems (*e.g.* Pd(0)/PCy_3_) lead to imino-Pd(ii) intermediates. In these cases, it is possible that SET oxidative addition is operative. The mechanistic divergence observed for different phosphine ligands during N–O oxidative addition is reminiscent of the substrate dependent mechanistic divergence often observed for oxidative addition of Pd(0) into aryl iodides *vs.* alkyl iodides (concerted *vs.* SET).^31^

**Scheme 15:**
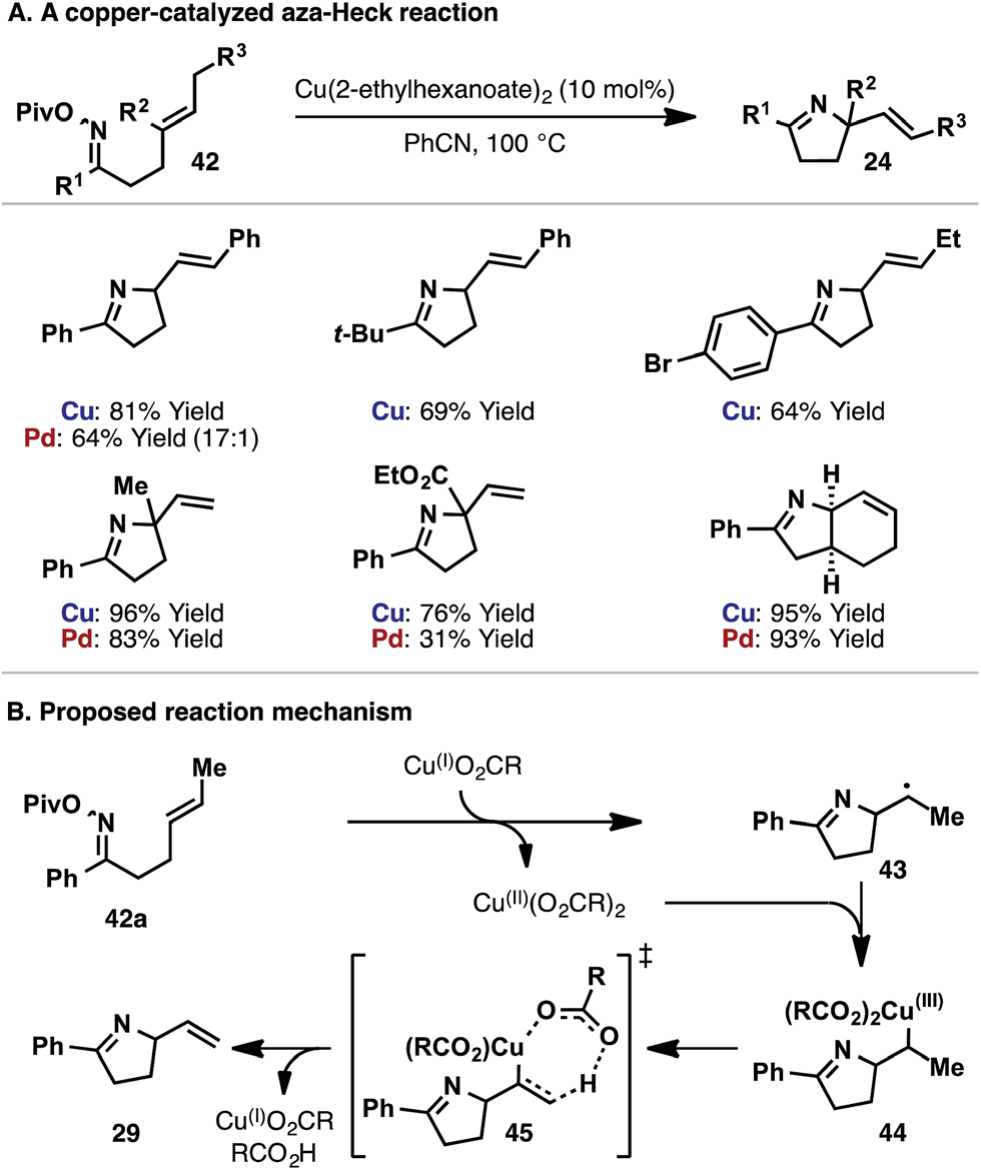
Copper-catalyzed aza-Heck-like cyclization *via* an iminyl radical.

In Scheme 14B, iminyl radical cyclization leads to products lacking an alkene. However, under copper-catalyzed conditions we have found that Heck-like products (*i.e.* those with an alkene) can be accessed using iminyl radical-based cyclizations (Scheme 15).^13b,33^ This finding is significant as it allows the replacement of a rare-earth transition metal catalyst with an isoelectronic first-row system. Our studies in this area were inspired, in part, by reports from Liebeskind and co-workers, who harnessed copper-based systems to activate the N–O bonds of oxime esters.^34^ Under optimized conditions, Cu(2-ethylhexanoate)_2_-catalyzed cyclization of a range of pivaloyl oxime esters **42** occurs in good to excellent yields (Scheme 15A). The reaction tolerates cyclic, 1,1-disubstituted and 1,2-disubstituted alkenes, demonstrating similar scope to the aforementioned Pd-catalyzed protocols. For comparative purposes, the yields obtained for Pd-catalyzed aza-Heck cyclization of the corresponding *O*-pentafluorobenzoyl oxime ester are included in Scheme 15A. Mechanistic studies suggest that Cu(i)-carboxylate (generated *in situ*) induces N–O homolysis of oxime ester **42a**, with the resulting iminyl radical cyclizing to alkyl radical **43** (Scheme 15B). Cu(ii)-carboxylate then traps **43** to generate alkyl-Cu(iii) intermediate **44**. As supported by studies from Kochi and co-workers,^35^ alkyl-Cu(iii) intermediate **44** then undergoes oxidative elimination, *via* transition state **45**, to afford product **29** with concomitant regeneration of the active Cu(i)-catalyst.

## New N–O donors: aza-Heck cyclizations of *N*-(pentafluorobenzoyloxy)sulfonamides and *O*-phenyl hydroxamates

**Scheme 16:**
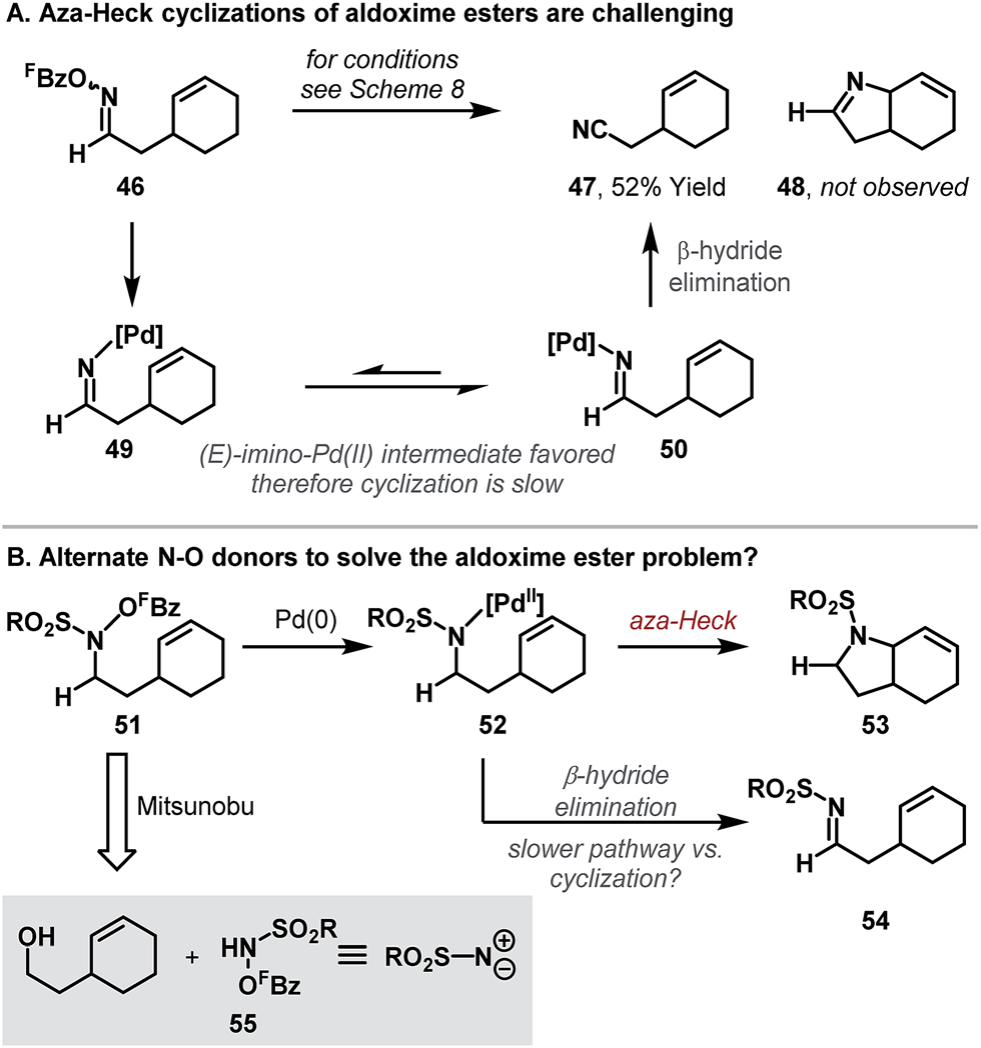
Development of alternate N–O bond donors for aza-Heck cyclizations.

A key limitation of the Narasaka–Heck protocol is that, in general, aldoxime esters are not suitable substrates. For example, in the attempted aza-Heck cyclization of **46**, target product **48** was not observed with nitrile side product **47** forming instead (Scheme 16A). Two key factors contribute to preferential formation of **47**: (a) access to reactive (*Z*)-imino-Pd(ii) intermediate **49** is not enforced by the small hydrogen substituent of aldoxime **46**, and (b) deleterious β-hydride elimination from sterically favored (*E*)-imino-Pd(ii) intermediate **50** is driven by the formation of the relatively stable nitrile **47**. To facilitate cyclizations of systems possessing hydrogen substituents adjacent to nitrogen, aza-Heck cyclizations of non-oxime derived N–O donors were sought (Scheme 16B). Specifically, cyclizations of *N*-pentafluorobenzoyloxysulfonamides **51** (to bicycle **53**) were considered appealing, because, at the stage of aza-Pd(ii) intermediate **52**, β-hydride elimination generates a less thermodynamically stable imine (**54**), such that this detrimental pathway was expected to be slower.^20^ A further benefit of the approach is that the substrates can be prepared directly by Mitsunobu reaction of the appropriate alcohol with *N*-(pentafluorobenzoyloxy)sulfonamide reagents, which we have found easy to prepare on multi-gram scale. As such, the overall sequence allows the two-step conversion of bis-homoallylic alcohols to chiral pyrrolidine targets.

**Scheme 17:**
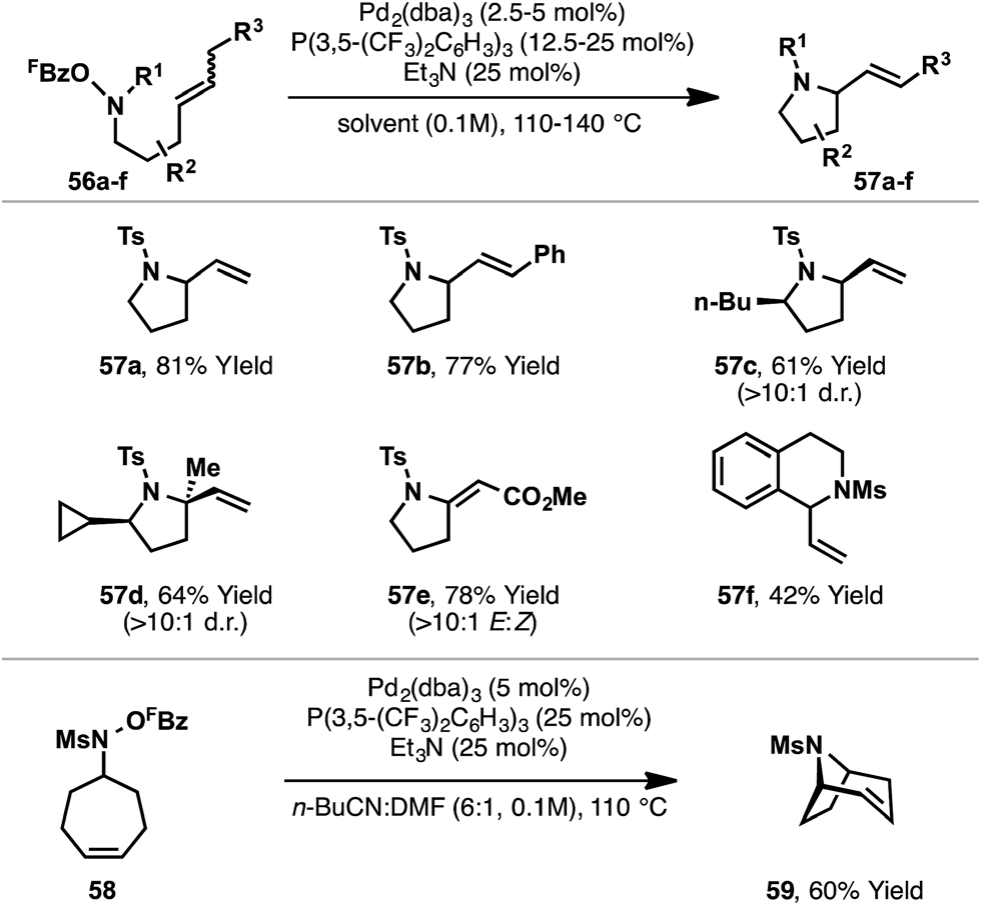
Aza-Heck cyclizations of *N*-(pentafluorobenzoyloxy)sulfonamides.

In the event, the approach outlined in Scheme 16B was successful, providing efficient access to a diverse array of heterocyclic ring systems (Scheme 17). The optimized catalyst system is the same as that described earlier, with fine-tuning of reaction solvent required on a case-by-case basis. Notable features of the process include: (a) selective β-hydride elimination away from nitrogen for cyclizations involving 1,2-disubstituted alkenes (*e.g.* **57a–b**), (b) excellent diastereoselectivities for cyclizations of substrates bearing substituents α to nitrogen (*e.g.* **57c–d**) and (c) the ability to promote challenging 6-*exo* cyclizations (*e.g.* **57f**). Furthermore, transannular cyclizations are also possible; cyclization of **58** provide tropane ring system **59** in high yield. Mechanistic studies support a reaction pathway analogous to the Narasaka–Heck reaction (see Scheme 7), with protodecarboxylation of the pentafluorobenzoate leaving group once again playing a key role.

Conceptually, the approach outlined in Scheme 16B is of broader interest because it uses sulfonamides **55** as bifunctional amino reagents. Specifically, the Mitsunobu step exploits the nucleophilic character of **55**, whereas the Pd-catalyzed aza-Heck step harnesses the electrophilic reactivity of the N–O bond. As such, further nucleophilic–electrophilic sequences could be envisaged with **55**, perhaps leading to a diverse array of methodologies. Studies towards this broad goal are ongoing.

**Scheme 18:**
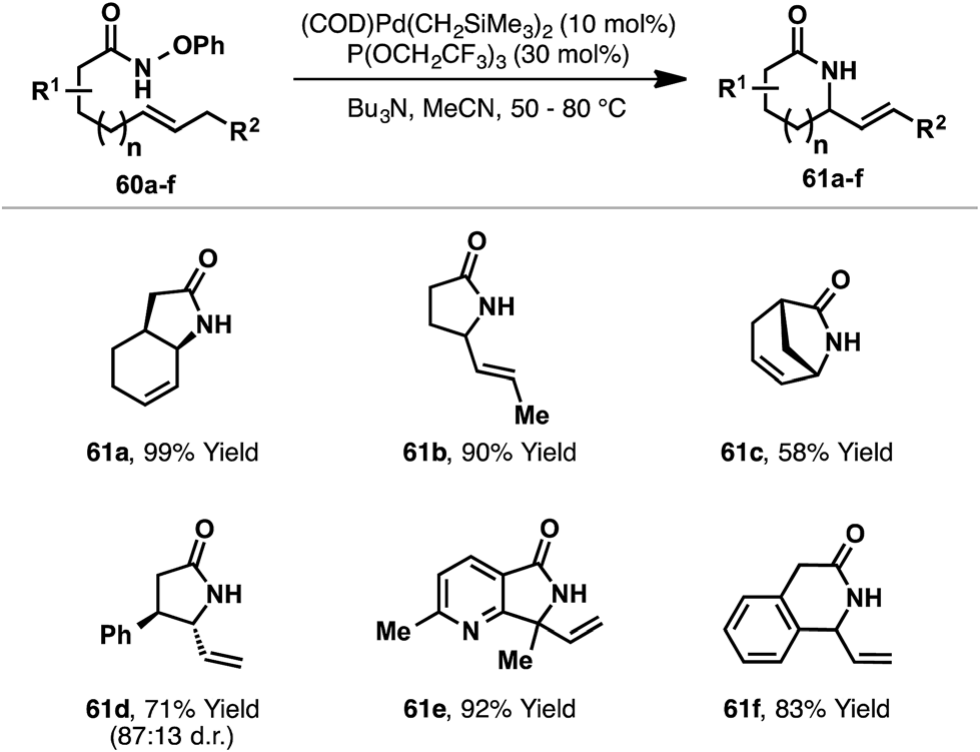
Aza-Heck cyclizations of *O*-phenyl hydroxamates.

Recently, Watson and co-workers reported a third distinct class of aza-Heck reaction, based on cyclizations of *O*-phenyl hydroxamates **60** (Scheme 18).^21^ The choice of an *O*-phenyl leaving group was crucial for efficient cyclization, as it suppressed competing Lossen rearrangement of **60**. Optimized conditions employ a Pd-system modified with the highly electron deficient phosphite P(OCH_2_CF_3_)_3_. Using this system, 5-*exo* cyclizations involving a wide range of di-, tri- and tetra-substituted alkenes were all effective, providing the targets in moderate to excellent yields. 6-*Exo* cyclizations are also achievable but require conformationally predisposed substrates (*e.g.* **61f**). Note that a secondary *O*-phenyl hydroxamate is required (R(CO)NHOPh), and N-alkylated systems are not tolerated. The proposed mechanism closely follows those outlined already, with oxidative addition of Pd(0) into the N–O bond of **60** occurring in advance of *syn*-stereospecific alkene amino-palladation; a series of experiments supported this latter proposition. In contrast to the processes described earlier, it is unclear whether or not this class of aza-Heck cyclization occurs *via* a cationic aza-Pd intermediate. Indeed, compared to pentafluorobenzoate, phenoxide is expected to dissociate much less readily, such that in the Watson process cyclization likely occurs in “neutral mode”.

The processes outlined in Schemes 17 and 18 could also potentially be achieved using an aza-Wacker approach, wherein the allylic amine targets are generated by Pd(ii)-catalyzed cyclization of an NH nucleophile with an alkene under oxidative conditions.^4^ Although often efficient, this approach suffers from several limitations. For example, few cyclizations of primary amides are known under aza-Wacker conditions (*cf.* Scheme 18),^36^ highlighting an inherent limitation of the method. With respect to aza-Wacker cyclizations involving NH-sulfonamides (*cf.* Scheme 17): large α-substituents are not tolerated (*cf.* **57d**), hindered acyclic olefins do not participate (*cf.* **57d**) and electron-deficient alkenes cannot be used due to competing conjugate addition (*cf.* **57e**).^37^ Furthermore, NH-sulfonamides required for aza-Wacker cyclization are not prepared directly from the alcohol because the requisite primary sulfonamides do not engage efficiently in conventional Mitsunobu reactions (*cf.* **55** to **51**). Additional advantages of the aza-Heck approach include predictable *syn*-migratory insertion of the alkene^37*c*^ and the ability to use tunable phosphine ligands (because oxidative conditions are avoided).

## Alkene 1,2-carboaminations *via* aza-Heck cascades

In all of the aza-Heck processes discussed so far, the alkyl-Pd(ii) intermediate formed after alkene migratory insertion undergoes β-hydride elimination to generate a new alkene. Further synthetic flexibility is potentially enabled by harnessing this intermediate for processes other than β-hydride elimination.^38,39^ Such an approach provides an appealing mechanistic framework for the development of synthetically valuable alkene 1,2-carboamination reactions.^40^

**Scheme 19:**
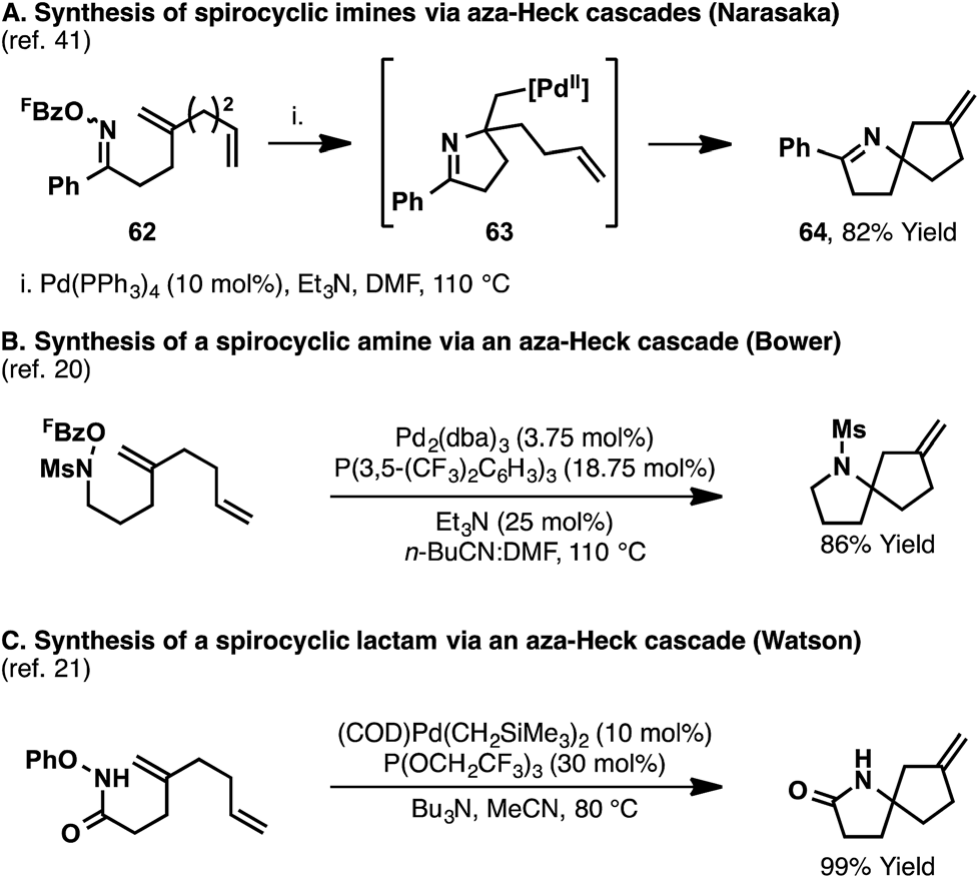
Synthesis of spirocyclic N-heterocycles *via* cascade aza-Heck cyclizations.

It has been shown that cascade aza-Heck reactions are possible by using specifically designed substrates, bearing multiple alkenes. In 2001, Narasaka reported a series of processes leading to spirocyclic imines (Scheme 19A).^41^ In one example, aza-Heck–Heck cyclization of oxime ester **62** generated spirocyclic system **64** in 82% yield. Initial imino-palladation of the 1,1-disubstituted alkene affords intermediate **63**, where β-hydride elimination is not possible. As such, a second migratory insertion can occur, this time involving the mono-substituted alkene, to deliver ultimately spirocycle **64**. Similar cascade reactions have been reported using more recently developed N–O donors (Scheme 19B and C).^20,21^

The processes in Scheme 19 involve intramolecular capture of the alkyl-Pd(ii) intermediate. Related intermolecular processes offer further flexibility. For example, our group has demonstrated carbonylative cascades involving a range of C-based or alcohol nucleophiles.^26^ Under a CO atmosphere, exposure of oxime ester **65** and Ph_4_B·NHEt_3_ to a P(3,5-(CF_3_)_2_C_6_H_3_)_3_ ligated Pd(0)-catalyst effected cyclization to target **66** in 62% yield. The proposed mechanism involves cyclization and carbonylation to afford cationic acyl-Pd(ii) intermediate **67**, which is intercepted by the pre-activated tetraarylborate (to give **68**), leading to product **66**. The choice of a tetraarylborate with a triethylammonium counterion was critical, with this latter component providing the proton required to trigger protodecarboxylation of the otherwise inhibitory pentafluorobenzoate leaving group (*cf.* Scheme 7). Note that in the oxime ester-based processes described earlier, the necessary proton is generated *via* the β-hydride elimination step. The carboamination protocol could be extended to other systems (*e.g.* **69a–e**), and alcohols and phenols were also found to be competent nucleophiles, leading to ester products (*e.g.* **69f**).^26,42^

Related carbonylative cascades involving alkynyl or vinyl boronates provide ynone and enones targets **70** and **71**, respectively (Scheme 21A). Here, the addition of a protic additive was not required, which suggests that the pentafluorobenzoate leaving group is sequestered as ^F^BzO-BPin, *via* Suzuki-like transmetallation from neutral acyl-Pd(ii) intermediate **72**. Similarly, under non-carbonylative conditions, aryl, vinyl or alkynyl boronates participated to provide 1,2-carboamination products **74a–f** from cyclization of oxime esters **73** (Scheme 21B).

**Scheme 20:**
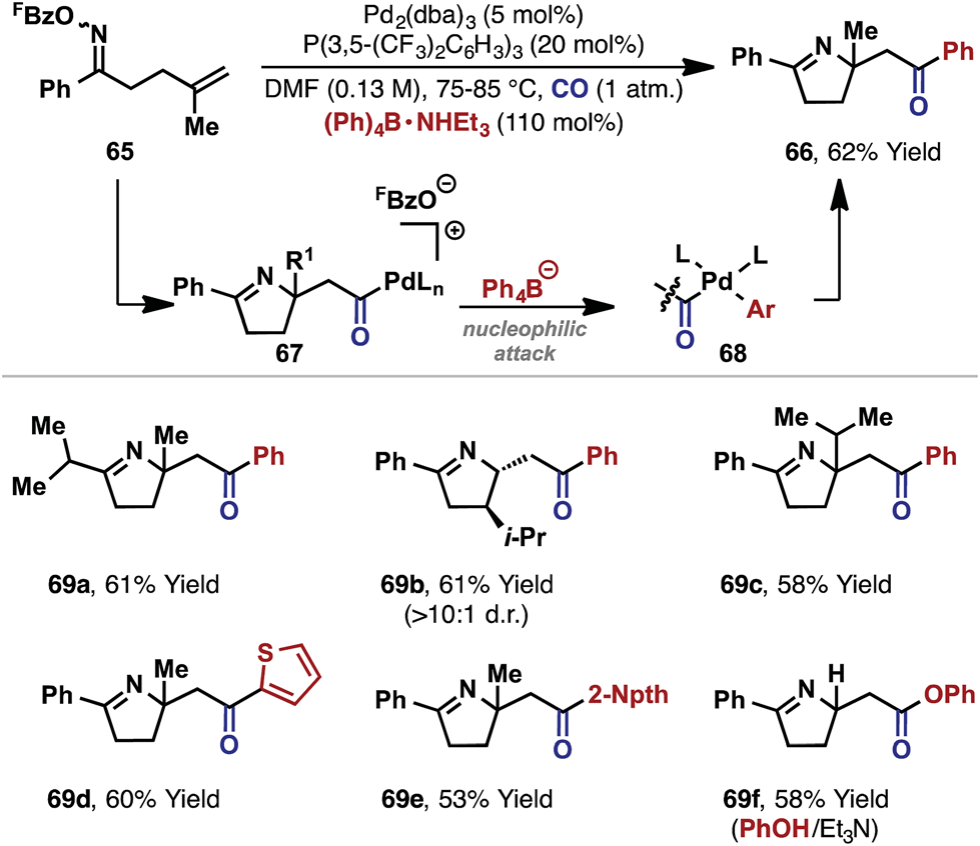
Carbonylative alkene 1,2-aminoacylation reactions.

**Scheme 21:**
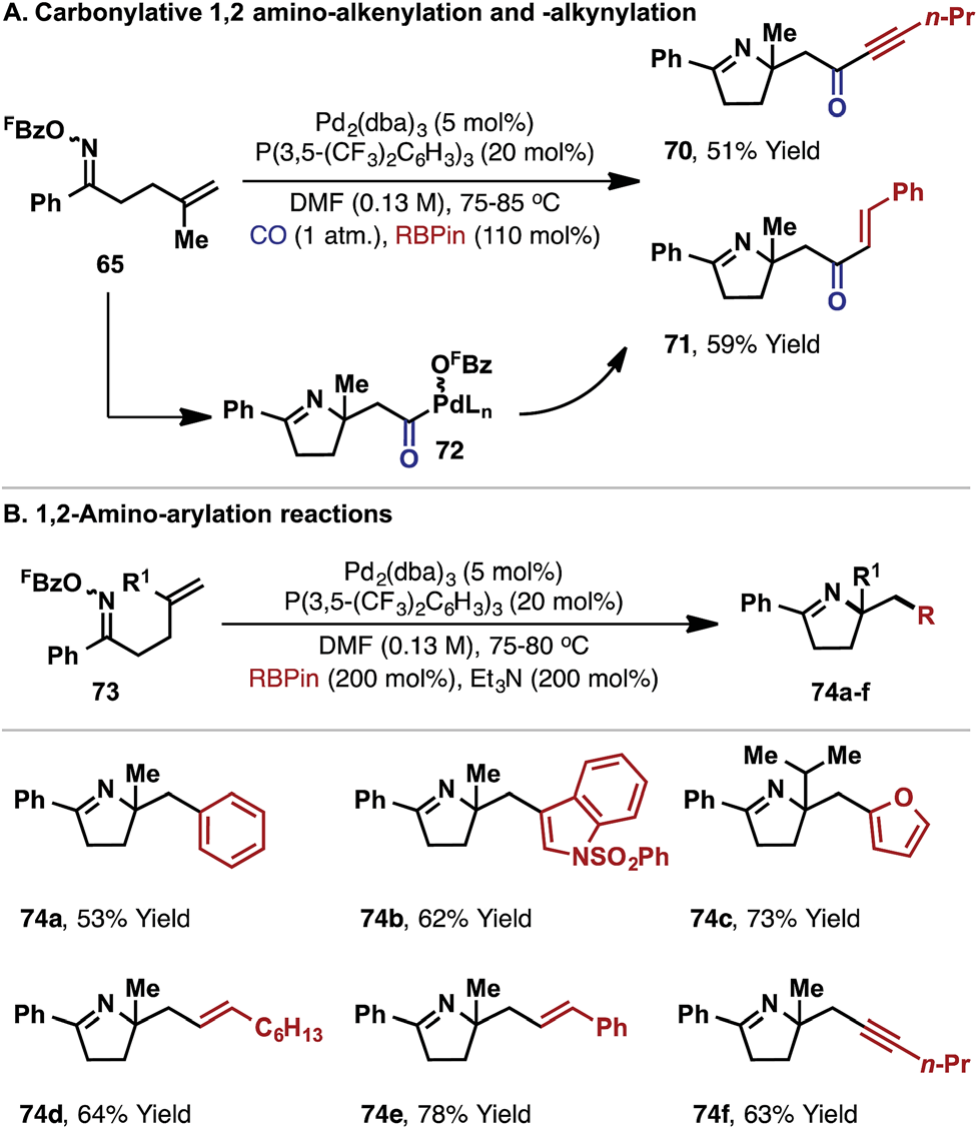
Further classes of 1,2-carboamination reaction.

The processes in Schemes 20 and 21 demonstrate that N–O oxidative addition can serve as the basis for a diverse suite of alkene 1,2-carboamination reactions. Specifically, 1,2-amino-acylation, -carboxylation, -vinylation, -arylation and -alkynylation reactions are all achieved within a unified mechanistic framework. Other redox-neutral Pd-catalyzed methods for alkene 1,2-aminoarylation exploit oxidative addition into the incoming C-based fragment,^40b,c,e,f^ and cannot access carbonylative products such as **66**. Conversely, carbonylative aza-Wacker cyclizations, which are oxidative processes, provide 1,2-aminocarboxylation products (*cf.* **69f**), but do not tolerate organometallic nucleophiles because these effect reduction of the active Pd(ii)-catalyst.^40*d*^ Accordingly, this manifold does not provide access to, for example, 1,2-aminoarylation products. Given the new aza-Heck N–O donors that have recently been developed (*vide supra*), it is likely that further variants of the “umpoled” alkene 1,2-carboamination strategy summarized here will emerge in the near future.

## Enantioselective aza-Heck reactions

**Fig. 1:**
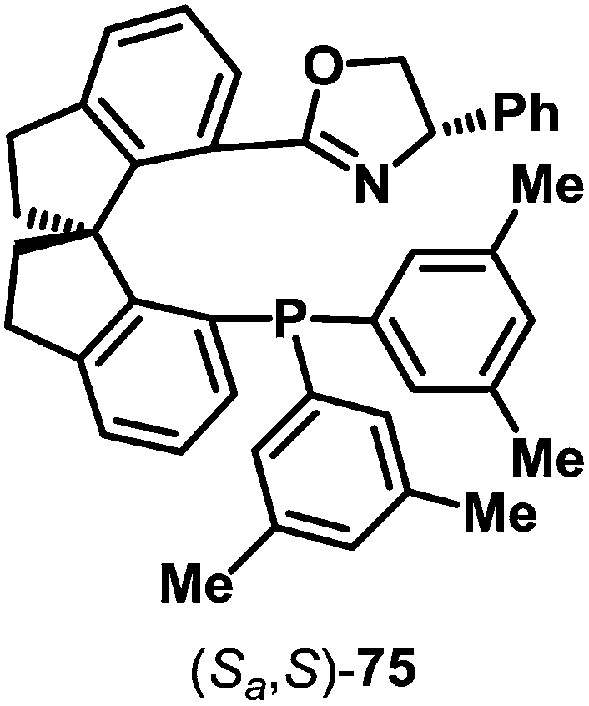
SPINOL-derived P,N-ligand (*S*_a_,*S*)-**75** for enantioselective aza-Heck cyclizations.

**Scheme 22:**
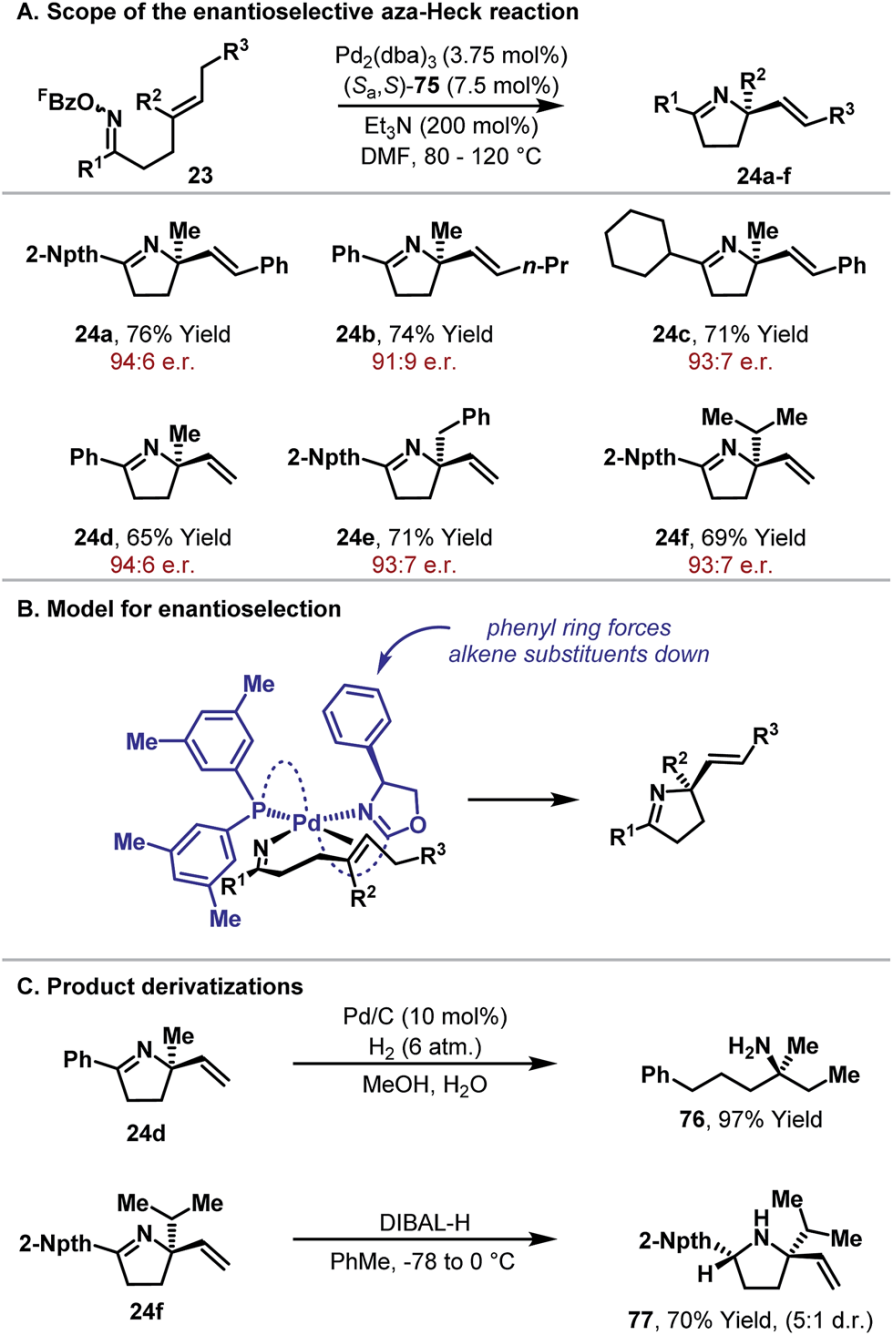
An enantioselective aza-Heck reaction.

From the discussion so far, it is clear that aza-Heck reactions provide convenient and flexible access to a diverse range of chiral N-heterocycles. Given the prevalence of stereodefined C–N bonds in natural products and pharmaceuticals, the development of enantioselective aza-Heck cyclizations became a key focus of our group. As highlighted above, aza-Heck cyclizations have relatively strict ligand requirements, such that the identification of effective chiral ligands was challenging. After extensive investigations, we found that SPINOL-derived P,N-ligand (*S*_a_,*S*)-**75** (Fig. 1) could promote highly enantioselective aza-Heck cyclizations of oxime esters with 1,1-disubstituted alkenes (Scheme 22).^43,44^ Presumably, the weakly donating oxazoline moiety is able to mimic the electronic effects of achiral electron-deficient triaryl phosphines used earlier (see Scheme 6B).

Using ligand (*S*_a_,*S*)-**75**, we found that enantioselective aza-Heck cyclization is efficient with a range of sterically diverse 1,1-disubstituted alkenes, generating targets **24a–f** in 68–86% yield and 91 : 9–95 : 5 e.r. (Scheme 22A). Notably, systems with large or small groups at R^2^ cyclized with similar levels of efficiency (*e.g.* **25f**, R^2^ = Me, 94 : 6 e.r. *vs* **24f**, R^2^ = iPr, 93 : 7 e.r.). A stereochemical model that accounts for this observation is outlined in Scheme 22B. The imino group resides *trans* to the oxazoline and the alkene coordinates *cis* to this unit. In this arrangement, the alkene favors an orientation where the –CH_2_R^3^ substituent is positioned away from the phenyl substituent of the oxazoline. In this model, there are minimal steric interactions between the R^2^ substituent and the chiral ligand, which accounts for the similar enantioselectivities observed for **24d**, **24e** and **24f**.

The enantioenriched dihydropyrrole products are suitable for derivatization *via* either the imine or alkene moieties (Scheme 22C). Exhaustive hydrogenation of **24d** provided chiral α-tertiary amine **76** in 97% yield, whereas DIBAL-H reduction of imine **24f** generated pyrrolidine **77** with good levels of diastereocontrol. The cyclizations in Scheme 22A are the first examples of highly enantioselective aza-Heck reactions, adding to a rare yet powerful class of processes that involve enantioselective migratory insertion of alkenes into Pd–N bonds.^4,8,45^ The development of further enantioselective aza-Heck cyclizations, including processes that use other N–O donors, is the focus of ongoing efforts.

## Conclusions

This perspective summarizes recent progress in the use of aza-Heck cyclizations for the synthesis of chiral nitrogen heterocycles. Building on Narasaka's seminal report, and aided by a detailed interrogation of reaction mechanism, highly efficient cyclizations of oxime esters with cyclic, 1,1- and 1,2-disubstituted alkenes have been developed. Our collective studies have highlighted key ligand requirements and provided a rationalization for the privileged role of pentafluorobenzoate leaving groups in such processes. This information guided the design of alkene 1,2-carboamination processes that are initiated by N–O oxidative addition of Pd(0)-catalysts. This fundamental mechanistic step is key to the work described here, and Narasaka's observation of this unusual process heralded the development of a wide range of N–O based processes outside the immediate area of aza-Heck chemistry. Indeed, imino-Pd(ii) intermediates now underpin a diverse array of redox-neutral processes including alkene 1,2-carboaminations,^26^ aryl C–H aminations,^9^ alkene aziridinations,^46^ alkene 1,2-iodoaminations,^47^ aryne aminofunctionalizations,^38^ and C–C bond activations.^25^ Exciting recent developments in aza-Heck chemistry have extended N–O oxidative addition to other N–O donors; this is an area that has great potential. Indeed, recent examples of highly enantioselective aza-Heck processes set the stage for the development of a family of redox-neutral enantioselective C–N bond forming processes that are initiated by oxidative addition at electrophilic nitrogen sources.
